# Frontal Cortex Segmentation of Brain PET Imaging Using Deep Neural Networks

**DOI:** 10.3389/fnins.2021.796172

**Published:** 2021-12-08

**Authors:** Qianyi Zhan, Yuanyuan Liu, Yuan Liu, Wei Hu

**Affiliations:** ^1^School of Artificial Intelligence and Computer Science, Jiangnan University, Wuxi, China; ^2^Jiangsu Key Laboratory of Media Design and Software Technology, Wuxi, China; ^3^Department of Nuclear Medicine, Nanjing Medical University, Affiliated Wuxi People's Hospital, Wuxi, China

**Keywords:** brain image segmentation, convolutional auto-encoder, conditional generative adversarial network, PET, Alzheimer's disease

## Abstract

^18^F-FDG positron emission tomography (PET) imaging of brain glucose use and amyloid accumulation is a research criteria for Alzheimer's disease (AD) diagnosis. Several PET studies have shown widespread metabolic deficits in the frontal cortex for AD patients. Therefore, studying frontal cortex changes is of great importance for AD research. This paper aims to segment frontal cortex from brain PET imaging using deep neural networks. The learning framework called **F**rontal cortex **S**egmentation model of brain **PET** imaging (FSPET) is proposed to tackle this problem. It combines the anatomical prior to frontal cortex into the segmentation model, which is based on conditional generative adversarial network and convolutional auto-encoder. The FSPET method is evaluated on a dataset of 30 brain PET imaging with ground truth annotated by a radiologist. Results that outperform other baselines demonstrate the effectiveness of the FSPET framework.

## 1. Introduction

Alzheimer's disease (AD) is a progressive disease that destroys memory and other important mental functions. As of 2019, it ranked as the sixth leading cause of death in China (Vos et al., 2020). There are more than 10 million patients with AD in China, a country with the most AD patients in the world (Jia et al., 2020).

AD is usually diagnosed based on the clinical manifestation. Nowadays, medical imaging including computed tomography (CT) or magnetic resonance imaging (MRI), and with single-photon emission computed tomography (SPECT) or positron emission tomography (PET), can be used to help doctors understand the pathophysiology of AD, for example, Aβ plaques, neurofibrillary tangles, and neuroinflammation. Moreover, the pathophysiology of AD is believed that starts years ahead of the of clinical observation, and helps detect AD earlier than conventional diagnostic tools (Marcus et al., 2014).

**Figure 1:**
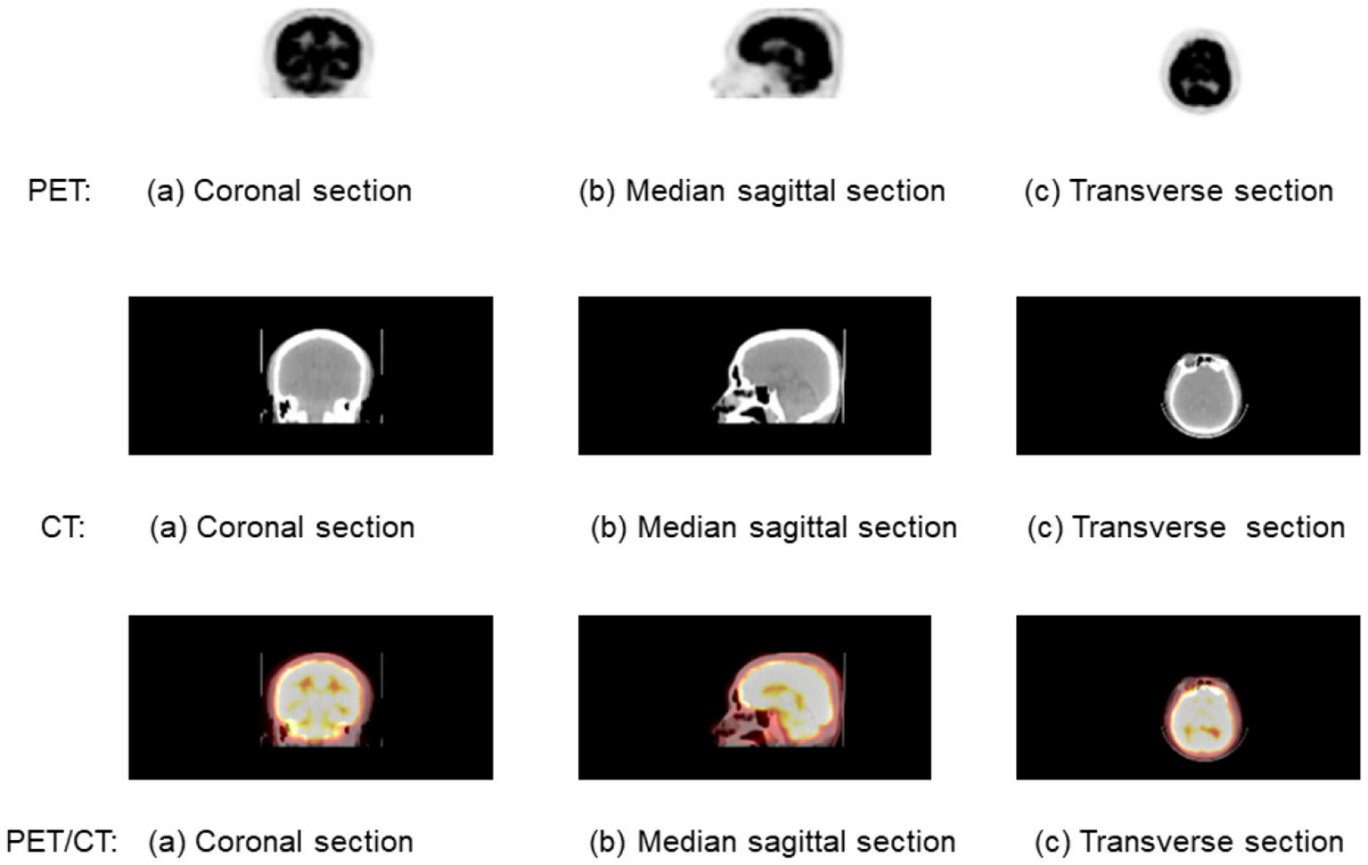
A brain positron emission tomography (PET)/computed tomography (CT) fusion image. The first line is the PET imaging, and the second line is the CT imaging. The fusion imaging of PET/CT is list in the third line. Each line from left to right is (a) coronal section, (b) median sagittal scan, and (c) transverse section.

Among the above medical imaging technique, PET/CT is a nuclear medicine technique that combines a PET scanner and a CT scanner to acquire sequential images from both devices in the same session, which are combined into a single superposed image. Figure 1 shows the brain PET/CT fusion image. The first line is the PET imaging, and the second line is the CT imaging. The fusion imaging of PET/CT is list in the third line. Each line from left to right is (a) coronal section, (b) median sagittal section, and (c) transverse section.

**Figure 2:**
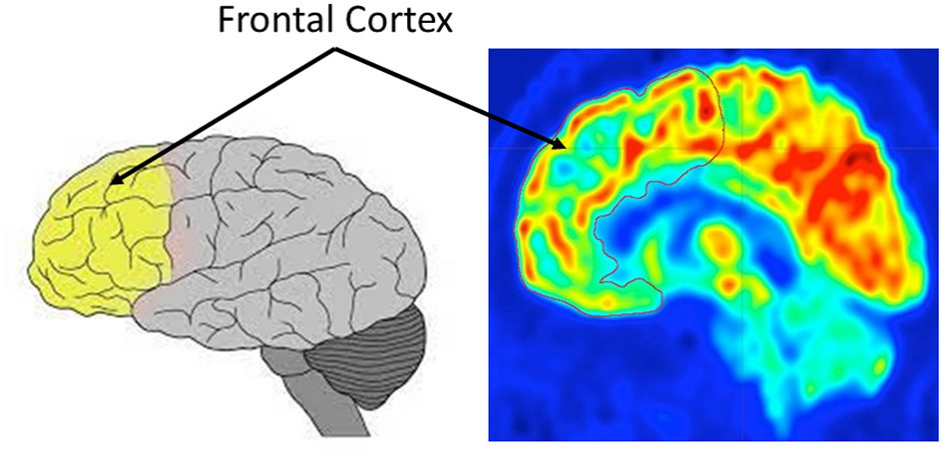
The frontal cortex in the brain: the left is anatomical location, and the right is for ^18^F-FDG positron emission tomography (PET) imaging.

^18^F-FDG PET imaging of brain glucose use and amyloid accumulation is a research criteria for AD diagnosis (Berti et al., 2011). Several ^18^F-FDG PET studies have been conducted to estimate AD-related brain changes. They have consistently shown widespread metabolic deficits in the neocortical association areas, such as frontal cortex. Further studies have demonstrated that CMRglc in frontal cortex suffers an average decline of 16−19% over a 3-year period (Smith et al., 1992; Mielke et al., 1994). Frontal cortex covers frontal lobe and contains most of the dopamine neurons. Figure 2 shows the location of frontal cortex in the brain. The yellow part of the left subfigure is its anatomical location, while the red contour in the right subfigure indicates its location in ^18^F-FDG PET imaging. Due to its sensitive to detect frontal cortex changes over time, ^18^F-FDG PET imaging can be used not only for AD diagnosis but also to monitor dementia progression and therapeutic interventions. Therefore, PET imaging are valuable in the assessment of patients with AD. Moreover, the frontal cortex segmentation of PET imaging is crucial for understanding AD progression on AD-related regions in brain.

Although frontal cortex segmentation is an important problem for AD research. However as far as we know, this paper is the first work that studies the frontal cortex segmentation problem for PET imaging. Unlike organ or tumor, which is different from other tissue with gray-level, texture, gradients, edges, shape, etc., frontal cortex is a part of brain without obvious boundaries. Moreover, supervised learning frameworks need segmentation ground truth from professional doctor, and it is difficult to get large number of annotated imaging. All these makes frontal cortex segmentation a tough problem.

Since manual segmentation is time consuming, automatic semantic segmentation for medical images, which makes pathological structures changes clear in images, becomes one of the hottest research topic in image processing. Currently, more and more machine learning technologies have been used in medical applications, such as medical single processing, medical image processing, medical data analyzing, and so on (Jiang et al., 2021a,b; Yang et al., 2021). Brain and brain tumor segmentation is one of the most popular medical image segmentation tasks (Szilagyi et al., 2003; Tu and Bai, 2009; Zhang et al., 2015; Jiang et al., 2019). Many approaches have been proposed to address this problem, such as thresholding (Sujji et al., 2013), edge detection (Tang et al., 2000), Markov random fields (MRF) (Held et al., 1997), and support vector machine (SVM) (Akselrod-Ballin et al., 2006).

Due to the rapid development of deep learning, neural networks, which can extract hierarchical feature of images, become one of the most effective technique in brain imaging segmentation (Fakhry et al., 2016; Işın et al., 2016; Zhao et al., 2018). U-net (Ronneberger et al., 2015) and its 3D version V-Net (Milletari et al., 2016) are the most well-known deep learning architecture in medical image segmentation. Recently, organ and tissue's shape and position priors are combined into the segmentation algorithm to improve the accuracy. (Oktay et al., 2017) proposes a training framework ACNN, which incorporates cardiac anatomical prior into CNN. Boutillon et al. (2019) combines scapula bone anatomical prior into a conditional adversarial learning method.

The related work has made large progress in semantic segmentation in medical imaging. However, they are not designed for frontal cortex segmentation in PET imaging, and they cannot be utilized directly for this problem. Motivated by this, in this paper, we propose the supervised segmentation framework: **F**rontal cortex **S**egmentation model of brain **PET** imaging (FSPET). The FSPET model based on both conditional generative adversarial network (cGAN) and convolutional auto-encoder (CAE) incorporates the anatomical prior to improve the prediction accuracy.

The contribution of FSPET dedicated to frontal cortex segmentation is threefold. First, the CAE is used to find the embedding of frontal cortex shape priors in latent space. Second, the segmentation method based on U-net, as the generator of cGAN, learns the feature of frontal cortex to generate the binary mask in PET imaging. Third, the anatomical prior is fused into the discriminator model in cGAN to get more precise prediction. Extensive experiments demonstrate the effectiveness of the proposed FSPET model.

## 2. Methods

**Figure 3:**
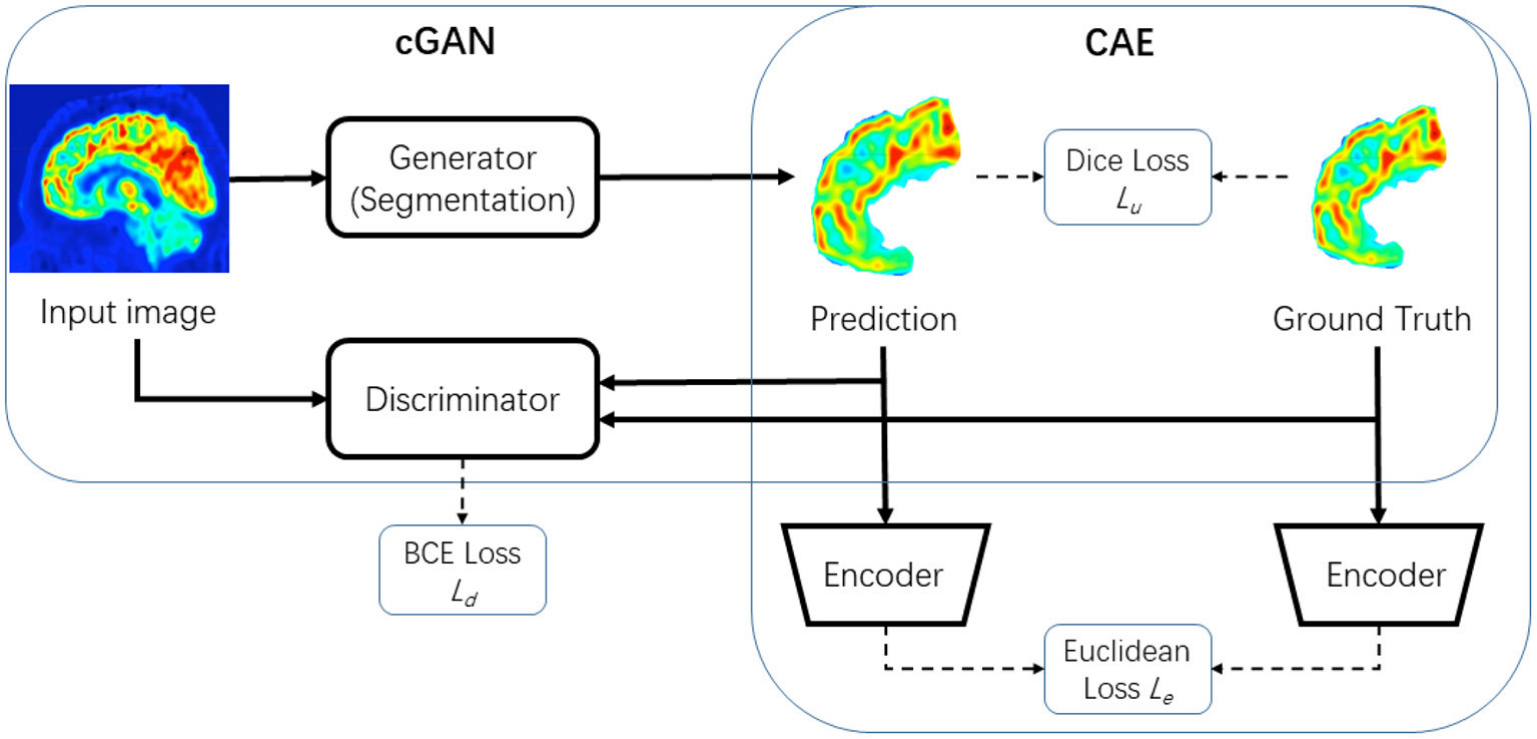
Framework of proposed model FSPET based on conditional generative adversarial network (cGAN) and convolutional auto-encoder (CAE).

In this section, we will introduce the proposed FSPET framework in detail, which combines the prior of frontal cortex shape in the deep neural networks, as shown in Figure 3. FSPET contains two parts: cGAN and CAE.

### 2.1. Conditional Generative Adversarial Networks

Generative Adversarial Network (GAN) (Goodfellow et al., 2014) is widely used for data augmentation by generating new images. Since PET imaging shows low contrast, low resolution, and blurred boundaries between different tissues, GAN is becoming a popular method for medical image segmentation (Luc et al., 2016; Son et al., 2017; Souly et al., 2017).

cGAN (Mirza and Osindero, 2014) is an extension of GAN, which is used as a machine learning framework for training generative models. The proposed FSPET model adopts the framework in Conze et al. (2021) based on cGAN, which consists of two neural networks: the generator G and the discriminator D.

**Figure 4:**
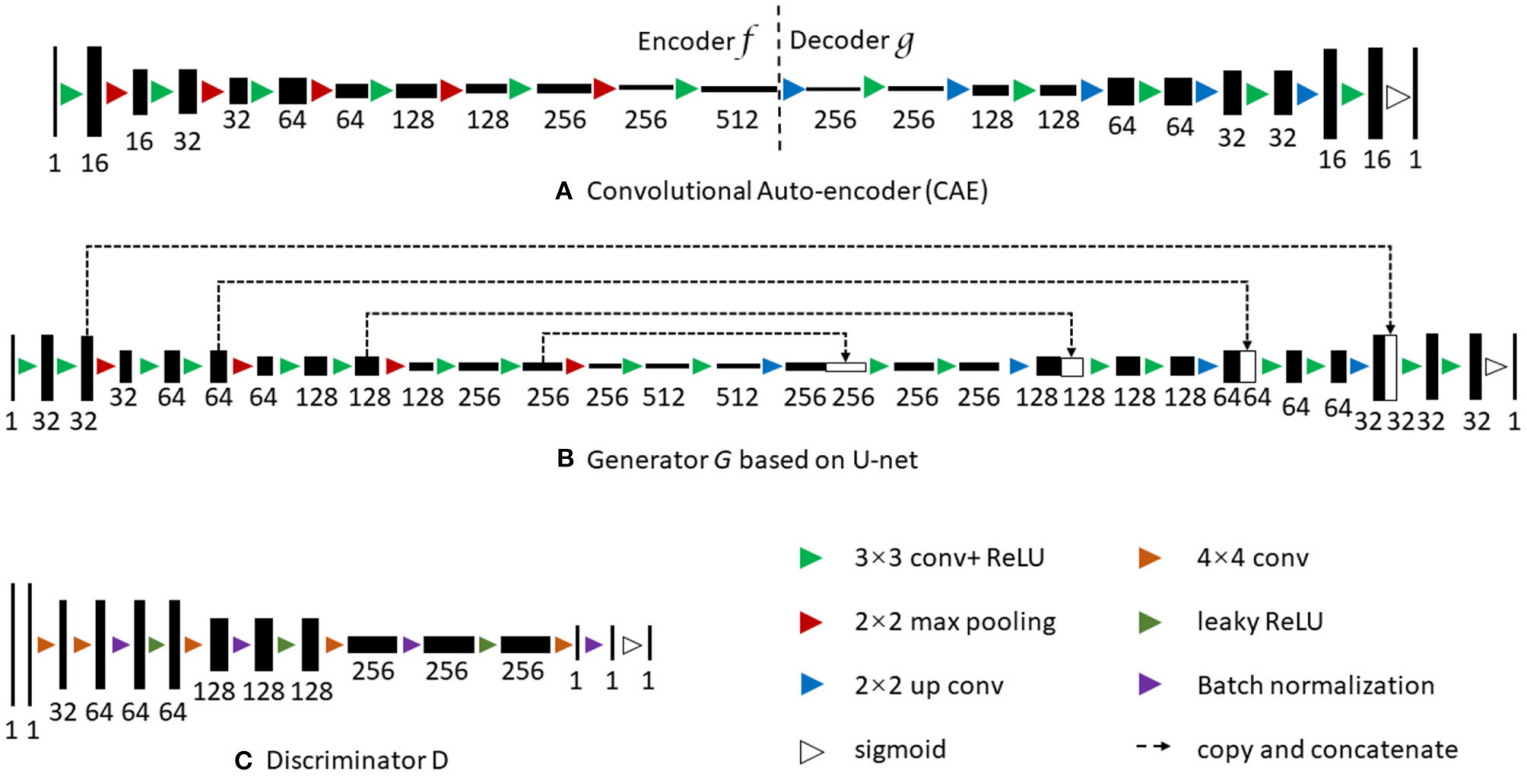
FSPET architecture: **(A)** convolutional auto-encoder (CAE), **(B)** Generator (G), and **(C)** Discriminator (D).

The generator G of cGAN in the FSPET model is the segmentation framework, which learns the feature of frontal cortex to generate the binary mask in PET imaging. Formally, let *x* be the source image and *y* be the ground truth image of class label $y_{i}\in L=\left\{1,2,\ldots ,c\right\}$. The generator learns the mapping between images and labels G:x→L by optimizing the loss function using stochastic gradient descent. The generator of cGAN is often based on U-net framework. The network consists of a contracting path and an expansive path (shown in Figure 4B). The contracting path is a convolutional network that consists of repeated application of 3 × 3 convolutions, each followed by a rectified linear unit (ReLU) and a 2 × 2 max pooling operation. Dice loss is used in U-net to compare the prediction *G*(*x*) and ground truth *y*, in which the loss function is as follows:


(1) $$\begin{array}{l} L_{u}={𝔼}_{x,y}\left[L_{dice}\left(G\left(x\right),y\right)\right]. \end{array}$$

The discriminator D in FSPET (shown in Figure 4C) inputs are source images and prediction to be evaluated. D distinguishes the given boundary by the generator from the realistic segmentation. The output is a binary prediction as to whether the image is real (class = 1) or fake (class = 0). In cGAN, binary cross entropy (BCE) loss is used to determine the loss function:


(2) $$\begin{array}{l} L_{d}={𝔼}_{x,y}\left[-log\left(D\left(x,G\left(x\right)\right)\right)\right]. \end{array}$$

### 2.2. Convolutional Auto-Encoder

Auto-encoder is a type of neural networks used to learn a representation (encoding) for a set of data. It imposes a bottleneck in the network, which forces a compressed knowledge representation of the original input. An auto-encoder consists of two parts, the encoder and the decoder, which can be defined as *f* and *g* such that:


$$\begin{array}{l} \;f:X\to \text{h}\\ \;g:\text{h}\to X\\ f,g=arg\;\underset{f,g}{min}||X-\left(f∙g\right)X||, \end{array}$$

where X is the input and **h** is usually referred to as code, the latent representation of the input. Motivated by Oktay et al. (2017), we utilized the CAE to find the embedding of frontal cortex shape priors in latent space (shown in Figure 4A). The BCE loss is minimized in the CAE framework with the ground truth *y* as input:


(3) $$\begin{array}{l} L_{CAE}\left(f,g\right)={𝔼}_{x,y}\left[-log\left(y,g\left(f\left(y\right)\right)\right)\right]. \end{array}$$

As shown in Figure 4A, after CAE is fixed, we use its encoder part *f* for segmentation training. By conducting CAE low-dimensional projection on both prediction and ground truth, we can minimize the loss function as:


(4) $$\begin{array}{l} L_{e}={𝔼}_{x,y}\left[{\left(f\left(G\left(x\right)\right)-f\left(y\right)\right)}^{2}\right]. \end{array}$$

### 2.3. Fusion

We have obtained three loss functions from different parts of the FSPET model respectively, whose information are listed in Table 1. Finally, we fuse the U-net segmentation method with frontal cortex shape priors. In the backward propagation, the loss function of generator G is:


(5) $$\begin{array}{l} \begin{array}{l} L_{G}\left(G,D,f\right)=\;L_{d}+\lambda_{1}L_{u}+\lambda_{2}L_{e}\\ \;=\;E_{x,y}\left[-log\left(D\left(x,G\left(x\right)\right)\right)\right]\\ \;+\;\lambda_{1}E_{x,y}\left[L_{dice}\left(G\left(x\right),y\right)\right]\\ \;+\;\lambda_{2}E_{x,y}\left[{\left(f\left(G\left(x\right)\right)-f\left(y\right)\right)}^{2}\right], \end{array} \end{array}$$

**Table 1:** Loss in the proposed FSPET model.

**Notation**	**Loss type**	**Evaluated by**	**Calculated from**	**Loss function**
*L*_*u*_	Dice	Prediction and ground truth	U-net G	*E*_*x, y*_[*L*_*dice*_(*G*(*x*), *y*)]
*L*_*d*_	BCE	Prediction and input	Discriminator D	*E*_*x, y*_[−*log*(*D*(*x, G*(*x*)))]
*L*_*e*_	Euclidean	Prediction and ground truth	encoder in CAE *f*	$E_{x,y}\left[{\left(f\left(G\left(x\right)\right)-f\left(y\right)\right)}^{2}\right]$

where λ_1_ and λ_2_ are the weighting factor. Minimizing *L*_*u*_ tends to provide rough frontal cortex shape prediction, while maximizing *log*(*D*(*x, G*(*x*))) is designed to improve contour delineations. At the same time, the latent loss *L*_*e*_ guarantees the global consistent and precise prediction similar to the original segmentation.

Additionally, the loss function of discriminator D is:


(6) $$\begin{array}{l} \begin{array}{l} L_{D}\left(G,D\right)=\;E_{x,y}\left[-log\left(D\left(x,y\right)\right)\right]\\ \;+\;E_{x,y}\left[-log\left(1-D\left(x,G\left(x\right)\right)\right)\right]. \end{array} \end{array}$$

It maximizes *log*(*D*(*x, y*), which is the loss between input and ground truth. Simultaneously, it minimizes loss value for generated −*log*(1 − *D*(*x, G*(*x*))) masks. The optimization proceeds in alternative periods on G and D using stochastic gradient descent.

## 3. Experiments

In this section, we conduct extensive experiments to validate the effectiveness of FSPET.

### 3.1. Validation Setup

*Dataset:* We collected 30 ^18^F-FDG PET images from different patients and their sensitive information was erased. The brain PET images were acquired using a PET/CT (Discovery STE, General Electric, Waukesha, USA) approximately 1 h after an intravenous injection of ^18^F-FDG (10 mCi). The original data were stored in Digital Imaging and Communications in Medicine (DICOM) format. Images were then resampled with a resolution 512 × 512 pixels. Frontal cortex in all images were annotated by a radiologist with 7 years experience to obtain the ground truth and also the shape priors.

*Baselines:* We compare FSPET with different baseline methods in frontal cortex segmentation for brain PET imaging. The comparison methods used in the experiments include:

- **U-net** (Ronneberger et al., 2015): U-net is the classical segmentation algorithm for medical images, and the generator G of FSPET is based on U-net. The architecture (shown in Figure 4B) contains contraction path and symmetric expanding path. The former path is used to capture the context in the image and the latter one is used to enable precise localization using transposed convolutions.
- **ACNN** (Oktay et al., 2017): It utilizes the auto-encoder and T-L network to combine anatomical prior knowledge into CNNs. These regularizers make predictions that are in agreement with the shape priors.
- **cGAN-Unet** (Singh et al., 2018): It is proposed for breast mass segmentation in mammography. The cGAN is used in the segmentation framework, in which the generative network learns the features of tumors and the adversarial network guarantees the contour to be similar to the ground truth.

*Measurements:* With the definition of true positive (TP), true negative (TN), false positive (FP), and false negative (FN), the following metrics are used to provide an overall assessment of all methods:

- Dice coefficient (dice): It is a similarity measure over prediction and ground truth. It ranges between 0 and 1.

$$DSC=\frac{2TP}{2TP+FP+FN}$$

- Jaccard index (Jaccard): It is another similarity metric with range [0, 1].

$$J=\frac{TP}{TP+FP+FN}$$

- Sensitivity: It is a measure of how well a test can identify true positives.

$$TPR=\frac{TP}{TP+FN}$$

- Specificity: It is a measure of how well a test can identify true negatives.

$$TNR=\frac{TN}{TN+FP}$$

- Hausdorff distance (HD): It is the greatest of all the distances from a point in one set to the closest point in the other set. HD measures how far two contours of prediction and ground truth are from each other. With A and B are the set of non-zero voxels in labels images, HD is defined as:

$$\begin{array}{l} \;HD\left(X,Y\right)=max\left(h\left(A,B\right),h\left(B,A\right)\right)\\ \text{where}\;h\left(A,B\right)=max_{a\in A}min_{b\in B}||a-b|| \end{array}$$

*Training Detail:* In this paper, we use Adam optimization (Kingma and Ba, 2014), which is a stochastic gradient descent method that is based on adaptive estimation of first-order and second-order moments. To train the FSPET model, the CAE with BCE loss is first optimized based on (3). With the learning rate of 0.01 for 30 epochs, the batch size is fixed at 32. Then the optimization of cGAN proceeds in alternative periods on G and D according to (5) and (6). In (5), the weight $\lambda_{1}=10^{-2}$ and $\lambda_{2}=10^{-4}$. With the learning rate of 10^−4^, batch size 32 and 30 epochs enjoyed the best performance.

### 3.2. Results

Quantitative metric and score values are provided in Table 2 for frontal cortex segmentation. When comparing U-net and ACNN, the dice score improves from 71.03% to 74.57%, and HD score decreases from 38.73 to 35.48. This demonstrates that extending U-net with CAE allows the model taking advantage of latent representation of shape priors. Moreover, significant improvements can be noticed using cGAN-Unet comparing with U-net (38.73 to 30.32 on HD), which indicates the appropriateness of embedding U-net into a cGAN pipeline. Combining CAE and cGAN networks, the proposed FSPET model discriminates more efficiently frontal cortex from surrounding structures by achieving the best score with regard to dice, Jaccard index, sensitivity, and HD. In particular, large gains in terms of Jaccard index (55.04–71.47%) and HD (38.73–28.05) are reported between U-net and FSPET.

**Table 2:** Quantitative assessment of U-net (Ronneberger et al., 2015), ACNN (Oktay et al., 2017), cGAN-Unet (Singh et al., 2018), and the FSPET model.

**Model**	**Dice**	**Jaccard**	**Sensitive**	**Specificity**	**HD**
U-net	71.03 ± 21.37	55.04 ± 20.43	72.29 ± 26.76	**98.22** **±** **2.12**	38.73 ± 30.46
ACNN	74.57 ± 18.34	59.45 ± 19.17	78.37 ± 23.85	97.24 ± 1.88	35.48 ± 27.83
cGAN-Unet	79.04 ± 19.59	65.34 ± 14.45	79.75 ± 21.53	97.18 ± 2.57	30.32 ± 29.12
FSPET	**83.37** **±** **15.98**	**71.47** **±** **15.32**	**81.97** **±** **20.88**	96.93 ± 2.27	**28.05** **±** **25.78**

*Bold results indicate the best scores*.

**Figure 5:**
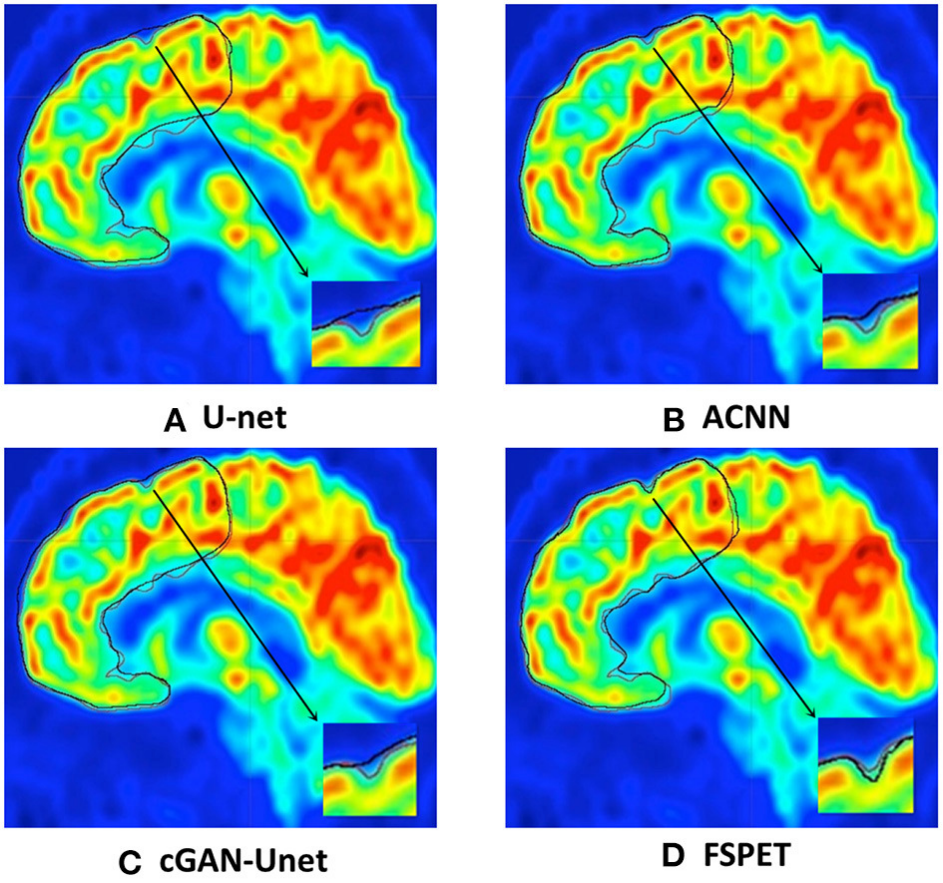
Frontal cortex segmentation in median sagittal section of brain positron emission tomography (PET) imaging using U-net, ACNN, cGAN-Unet, and the FSPET model. Ground truth and predicted contour are in red and black, respectively. **(A)** U-net. **(B)** ACNN. **(C)** cGAN-Unet. **(D)** FSPET.

Qualitative results for frontal cortex segmentation in median sagittal section of brain PET imaging are displayed in Figure 5. Compared to U-net, ACNN, and cGAN-Unet, which are prone to under- or over-segmentation, sometimes combined with unrealistic shapes, better contour adherence and shape consistency are reached by the FSPET model. We also take one example (shown in bottom right of each subfigure) for comparison, and the FSPET captures more complex shape and subtle contours compared to other frameworks. This reveals the importance of combining both adversarial networks and shape priors in the segmentation task.

## 4. Conclusion

This paper propose a deep learning framework to segment frontal cortex from brain PET imaging. The model based on both cGAN and CAE incorporates the anatomical prior to improve the prediction accuracy. Future work will utilize the proposed method to detect other parts of AD-related brain area, such as hippocampus.

## Data Availability Statement

The original contributions presented in the study are included in the article/supplementary material, further inquiries can be directed to the corresponding author/s.

## Ethics Statement

The studies involving human participants were reviewed and approved by Jiangnan University. The patients/participants provided their written informed consent to participate in this study.

## Author Contributions

QZ contributed to the conception of the study and wrote the manuscript. YuanyuanL performed the experiment. YuanL helped to perform the analysis with constructive discussions. WH contributed significantly to data preparation.

## Conflict of Interest

The authors declare that the research was conducted in the absence of any commercial or financial relationships that could be construed as a potential conflict of interest.

## Publisher's Note

All claims expressed in this article are solely those of the authors and do not necessarily represent those of their affiliated organizations, or those of the publisher, the editors and the reviewers. Any product that may be evaluated in this article, or claim that may be made by its manufacturer, is not guaranteed or endorsed by the publisher.
